# Investigation of the Influence of Hypercapnia on the Physiology of Ovigerous West Coast Rock Lobsters, *Jasus lalandii*, and Their Embryonic Development

**DOI:** 10.3390/biology14020132

**Published:** 2025-01-27

**Authors:** Annika Ritter, Christopher R. Bridges, Lutz Auerswald

**Affiliations:** 1 Institute of Metabolic Physiology/Ecophysiology, Heinrich-Heine University, D-40225 Düsseldorf, Germany; annika.c.ritter@gmail.com (A.R.); bridges@tunatech.de (C.R.B.); 2 Branch Fisheries Management, Department of Forestry Fisheries and the Environment (DFFE), Roggebaai, Cape Town 8012, South Africa; 3 Department of Animal Sciences, Stellenbosch University, Stellenbosch 7602, South Africa

**Keywords:** hypercapnia, ocean acidification, upwelling, acid–base regulation, embryonic development, ovigerous, physiology, maternal care

## Abstract

The West Coast rock lobster, *Jasus lalandii*, is a key ecological species and provides an important fishery resource in South Africa and Namibia. It is found along the west coast of southern Africa in the dynamic Benguela Current upwelling system. The low seawater pH of this system is expected to decline further due to ocean acidification and increase in upwelling in terms of frequency and severity. The lobster has therefore to respond to frequent and rapid changes in pH and other environmental impacts that are predicted to become more adverse in future. Although responses to such conditions are known for mature male and juvenile lobsters, there is a lack of information on mature females and later embryonic development. We addressed this by analysing the sensitivity of ovigerous (“berried”) female WCRLs and their eggs/embryos to hypercapnia (high pCO_2_, low pH) and formulated the following research questions: (1) Can berried female WCRLs respond swiftly to large changes in pH? (2) What physiological mechanisms facilitate a potential response to a rapidly declining pH, i.e., acute hypercapnia? (3) Does a potential response persist during prolonged hypercapnia? (4) Are eggs/embryos impacted by hypercapnia? To investigate this, we exposed berried WCRLs to acute (pH 7.5) and chronic (up to 60 days at pH 7.5 and 7.8) hypercapnia. We applied extracellular acid–base analysis, microscopic examination of egg growth and development, and SEM of female exoskeleton structure and egg membranes. The results revealed that berried females efficiently respond to acute and chronic hypercapnia by means of increasing bicarbonate concentrations in the haemolymph. Moreover, embryo growth and development are not impacted by chronic hypercapnia, but growth shows geographical area-specific differences. We conclude that females and embryos of *J. lalandii* are as resilient to hypercapnia as previously shown for males and juveniles.

## Simple Summary

The West Coast rock lobster (WCRL), *Jasus lalandii*, inhabits the dynamic Benguela Current upwelling system along the west coast of southern Africa and supports an important fishery. WCRLs have to respond to frequent and rapid changes in pH and other environmental impacts that are predicted to become worse. Responses to such conditions are known for mature male and juvenile lobsters, but not for mature females or embryonic development. We therefore analysed the sensitivity of ovigerous (“berried”) female WCRLs and their eggs/embryos to acute and chronic hypercapnia (high pCO_2_, low pH). We applied extracellular acid–base analysis, stereomicroscopic examination of egg growth and development, and SEM of female exoskeleton structure and egg membranes. Berried females efficiently respond to acute and chronic hypercapnia by means of increasing bicarbonate concentrations in the haemolymph. Embryo growth and development are not impacted by chronic hypercapnia, but growth shows geographical differences. We conclude that females and embryos of *J. lalandii* are as resilient to hypercapnia as previously shown for males and juveniles of the same species.

## 1. Introduction

**Figure 1:**
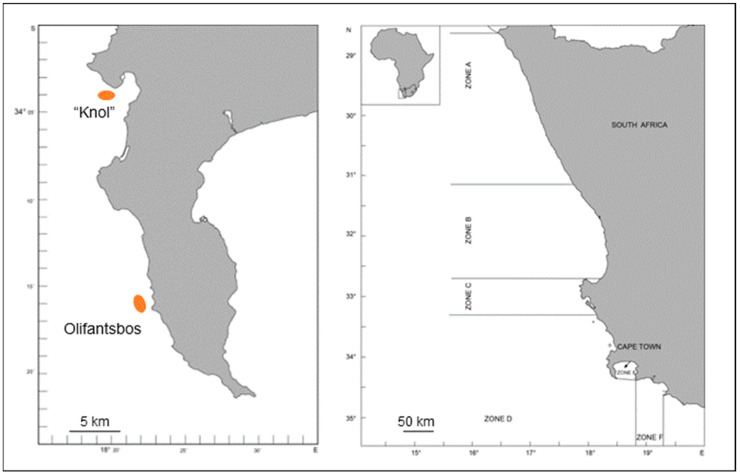
West Coast rock lobster capture area, showing the two sampling sites “Knol” in Hout Bay and Olifantsbos in fishing Zone D.

The West Coast rock lobster (WCRL), *Jasus lalandii*, is a slow-growing palinurid species from the southern African Atlantic coast that inhabits cold to temperate waters. The fishery of the WCRL is one of the most important in South Africa due to its high economic value and directly and indirectly employs about 4300 people in marine- and land-based employment [1]. The Branch: Fisheries Management of the respective national governmental department (currently Department of Forestry, Fisheries and the Environment—DFFE) manages the West Coast rock lobster (WCRL) resource per zone (Figure 1) by means of total allowable catch (TAC), by additional effort control, a minimum legal size, and a ban on retaining ovigerous (“berried”) females [2].

The WCRL is found mainly along the west coast of southern Africa in the Benguela Current System (BCLME), which is characterised by frequent upwelling events that result in hypercapnia in the range of pH 7.4–7.6 compared with normocapnic conditions of around pH 7.9–8.1 [3,4,5]. In addition, low-oxygen events caused by the decay of algal blooms and bacterial respiration can in some areas cause the water pH to drop to levels as low as 6.6 for several days [4]. In the long term, a further decline in pH is expected due to ocean acidification and increase in upwelling in terms of frequency and severity [6]. The rock lobster therefore has to respond to frequent and often rapid changes in pH and other environmental impacts such as rapid temperature changes and variations in oxygen levels. Responding to rapid environmental changes is not uncommon for crustaceans, however, which populate a variety of habitats and are therefore naturally exposed to a wide range of environmental parameters, resulting in a certain degree of physiological plasticity [7,8].

Previous research has revealed that *J. lalandii* is well adapted to this highly changeable habitat: the species is capable of efficiently maintaining its acid–base balance when exposed to acute and chronic hypercapnia [9,10]. This is achieved by a rapid increase in the bicarbonate levels in the haemolymph and is reversible. Bicarbonate buffering protects the pH-sensitive oxygen transport capacity of haemocyanin under hypercapnic conditions and provides optimum pH conditions for oxygen binding in the presence of a strong Bohr effect. Moreover, the oxygen affinity of haemocyanin is enhanced by an intrinsic modification of its molecular structure [9]. The immune response of this species is also very efficient and not impacted following chronic hypercapnia [11].

Mature female WCRLs moult in late austral autumn to early winter and mate shortly thereafter [12], when the female exoskeleton is still relatively soft and pleopodal setae are fresh for egg attachment [13,14]. In contrast to males, which use 99% of energy reserves accumulated during intermoult for growth, females use 95% for reproduction [15]. As a result, females grow slower than males. The fecundity of female *J. lalandii* varies along the Cape West Coast, with greater fecundity (more eggs) produced in areas where somatic growth is rapid [16]. Between the two sites investigated here, fecundity differs from approximately 104,000 to 137,000 eggs per lobster in the 75–79 mm carapace length class, a difference of 32% [16]. In times of nutritional stress, *J. lalandii* females sacrifice growth to optimize egg production [17]. Embryonic development occurs externally with the eggs attached to the ovigerous female’s pleopods (“berry” stage), which exposes them to ambient conditions.

Crustacean eggs rely exclusively on the maternal provision of biomaterials and metabolites (such as vitellogenin, lipoproteins, carotenoids, carbohydrates, amino acids, nucleotides, and nucleic acids), rendering the eggs “self-sufficient capsules” [18]. This is also true for *J. lalandii*, where the developing embryo relies exclusively on its yolk for energy and structural material until hatching as naupliosoma larvae after about 70–90 days [19,20]. Lipids and proteins accumulate concomitantly in adult crustacean ovaries during maturation and growth, and the GSI (gonadosomatic index) increases [18]. This too has been shown for WCRL ovaries [21,22]. Female lipid and protein accumulation success therefore results in optimal ovary development and translates directly into a successful maternal export to the larvae [14,18].

Early life stages of marine invertebrates are assumed to be exceptionally sensitive to environmental stress and may represent a bottleneck in species success [23]. The hypothesis of embryo sensitivity comes from the assumption that hypercapnic stress may lead to a higher metabolic demand (for example, for acid–base regulation) and depletion of the finite energy stores (see above) prior to hatching [24,25]. This in turn would compromise the hatching process and species fitness. However, although crustacean embryos and larvae are potentially susceptible to climate change stressors, information about the vulnerability to stressors in early ontogenetic stages is still lacking for many species [7,26,27].

Changes in environmental conditions in the habitat due to climate change can impact sexual reproduction. Identification of the most sensitive stages in reproductive cycles will therefore enable better prediction of a species‘ response to environmental change [28]. However, in this regard, only mature male and juvenile WCRLs have previously been investigated (see above). Impacts of hypercapnia on females and sensitivity of embryonic development are currently a knowledge gap for *J. lalandii* and palinurids in general.

We addressed this shortfall in the present study by analysing the sensitivity of ovigerous female WCRLs and their eggs to hypercapnia. Accordingly, we formulated the following research questions: (1) Can berried female WCRLs respond swiftly to large changes in pH? (2) What physiological mechanisms facilitate a potential response to a rapidly declining pH, i.e., acute hypercapnia? (3) Does a potential response persist during prolonged hypercapnia? (4) Are eggs/embryos impacted by hypercapnia?

## 2. Materials and Methods

### 2.1. Experimental Animals

All female West Coast rock lobsters (WCRLs), *Jasus lalandii*, were caught at two sites on the Atlantic coast of South Africa (Figure 1) by large, baited lobster traps at depths of approximately 80–200 m during the spawning season in the austral winter (August). The Knol station (34°04.50′ S 18°19.00′ E) is located inside an area where fast somatic growth has been observed, whereas the Olifantsbos station (34°13.00′ S 18°19.00′ E) is within a slow somatic growth area. A total of 63 berried WCRLs were captured, of which 42 of a mean carapace length (CL) of 69.5 ± 2.2 mm were caught at the Knol station and 21 lobsters of a CL of 64.7 ± 2.0 mm at the Olifantsbos station. Aboard the ship, berried females (i.e., carrying eggs) were transferred to a flow-through system until docking at the harbour of Cape Town (after approximately two days). Lobsters were transported cool and moist to the research aquarium of the Department of Forestry, Fisheries and the Environment (DFFE) within 30 min. Here, they were kept in 1500 L tanks with a flow-through system (flow rate ~430 L h^−1^) provided with aeration for one week until experimentation started (ambient seawater temperature (T_A_) ranged from 9 to17 °C, salinity 34.5–35.0‰). They were fed weekly with pieces of frozen sardines (*Sardinops sagas*) *ad libitum*.

### 2.2. Acute Response to Hypercapnia

Nine berried female lobsters of similar size from the Olifantsbos area were selected from the holding tanks and transferred to a 1000 L tank, where they were kept for 24 h prior to experimentation at water conditions given in Table 1 to acclimatise to experimental temperatures. Following these 24 h, a 0.5 mL haemolymph sample was taken from each lobster (see below), and four of the lobsters (CL: 69.5 ± 1.2 mm) were transferred to individual compartments inside two glass tanks (141 L) with normocapnic conditions (pH ~8.0), and the remaining five (CL: 69.6 ± 3.3 mm) were transferred to another two glass tanks with the same dimensions and compartments, but filled with hypercapnic seawater (pH ~7.5). The normocapnic pH represented that of the incoming seawater on this day and is close to the level during non-upwelling periods in the subtidal zone. In order to minimize the time difference in exposure to the set seawater parameters of the respective treatments, consecutive lobsters were placed in alternating treatments after the initial haemolymph sample (0 h) was withdrawn (i.e., first hypercapnia, second normocapnia etc.). After 1.5, 3, 5, 8, and 24 h, 0.5 mL pre-branchial haemolymph was withdrawn through the arthrodial membrane at the base of the fifth pair of pereiopods by syringe with a hypodermic needle (Neomedic 1 mL, 29 G), avoiding tail flips (i.e., accumulation of L-lactate) by securing the abdomen with a firm grip. Circulation in the tanks was maintained by JVP-202 12,000 L h^−1^ propellers (JVP, Ningbo, China) and air was provided from the aquarium’s compressed air system. The pH of the hypercapnic tank was set using a pH controller and the CO_2_ storage tank connected to a solenoid valve and a pH electrode (TUNZE, Penzberg, Germany) whereby CO_2_ was bubbled into the seawater as described previously [10]. Seawater pCO_2_ and [HCO_3_^−^ + CO_3_^2-^] were calculated using measured pH, salinity, and T_A_ and A_T_ [29] as constants in CO2SYS_v2.1. software [30]. Oxygen concentration was determined using a Multi 350i meter set (WTW, Weilheim, Germany). Water quality was monitored by measuring NH_3_ concentration (ammonia test kit, Sera, Heinsberg, Germany) and never exceeded 0.3 mg L^−1^.

### 2.3. Chronic Response to Hypercapnia

A systematic experimental design of a flow-through system according to guidelines by Riebesell et al. [31] (Figure 6.7–A-3) and Cornwall and Hurd [32] (Figure 3d) was essentially used: seawater from a subtidal area of Table Bay in front of the facility was continuously pumped into the aquarium facility and after filtration stored in the main water storage tank of the aquarium. From here, seawater flowed to replicate mixing tanks, where pH was adjusted. Seawater subsequently flowed to culture tanks (= sub-replicates). The normocapnic treatment used incoming pH conditions (ca. pH 8, Table 1), and the hypercapnic treatments were controlled in such a way that pH 7.8 and 7.5, respectively, were maintained in the culture tanks. The pH control systems as described above were used to reduce pH to ~7.8 and ~7.5 respectively. Specimens were separated into two groups for taking exclusively haemolymph or egg samples. Ovigerous females from both areas were individually incubated in four replicates for each of three treatments per sampling location to investigate embryonic development. Lobsters were from the Knol area (n = 12, CL = 69.5 ± 2.3 mm, w = 194 ± 6 g) and from the Olifantsbos area (n = 12, CL = 64.7 ± 2.1 mm, w = 180 ± 6 g). Only lobsters (n = 30) from the Knol area (CL = 72.5 ± 2.0 mm, w = 202 ± 6 g) were chosen for analysis of haemolymph chemistry after 60 days of incubation. In the respective two hypercapnic treatments, lobsters were acclimatized by gradually reducing the pH within three days. Lobsters were kept in three replicates per treatment (4, 3 and 3 WCRLs) and fed *ad libitum* weekly with black mussels (*Chromomytilus meridionalis*, *Mytilus galloprovincialis*). Haemolymph sampling after 60 days of exposure was carried out as described above. To minimise tank/placement effects, replicate tanks and culture tanks were routinely alternated. No mortalities occurred throughout the experiment.

### 2.4. Haemolymph Acid–Base Balance

Haemolymph pH was measured within 20 s after sampling using an Orion 3 star pH meter equipped with an Orion 8220 BNWP micro pH electrode (Thermo Scientific, Waltham MA, USA). Calibration was performed with NBS precision buffers (AppliChem, Darmstadt, Germany) at the same temperature as that of ambient seawater of the lobster tanks. To measure total CO2 (cCO_2_), a haemolymph subsample (100 μL) was immediately injected into a degassing (magnetic stirrer) chamber containing 200 μL of 100 mM H_2_SO_4_ and the liberated total CO_2_ (cCO_2_) determined by CO_2_ analyser (SBA4, PP Systems, Amesbury, MA, USA) using CO_2_-free N_2_ (technical) as carrier gas (50 mL min−1) calibrated against freshly made Na_2_HCO_3_ standards (1–10 mM). From these measured parameters (pH and cCO_2_), pCO_2_, and [HCO_3_^−^ + CO_3_^2−^] were calculated using derivatives of the Henderson–Hasselbalch equation, using constants derived from Truchot [33]. Concentrations of Ca_2_^+^, Mg_2_^+^, and L-lactate were determined using commercial kits (Diaglobal, Berlin, Germany; Roche, Penzberg, Germany) on small subsamples from each lobster. Haemocyanin (at 335 nm) and protein (at 280 nm) concentrations were measured spectrophotometrically in 1:50-diluted haemolymph vs. *J. lalandii* Ringer solution (0.52 M NaCl, 0.015 M MgSO_4_, 0.013 M CaSO_4_, 0.005 M KCl, 0.005 M NaHCO_3_, pH 7.8) [9].

### 2.5. Embryonic Development

**Figure 2:**
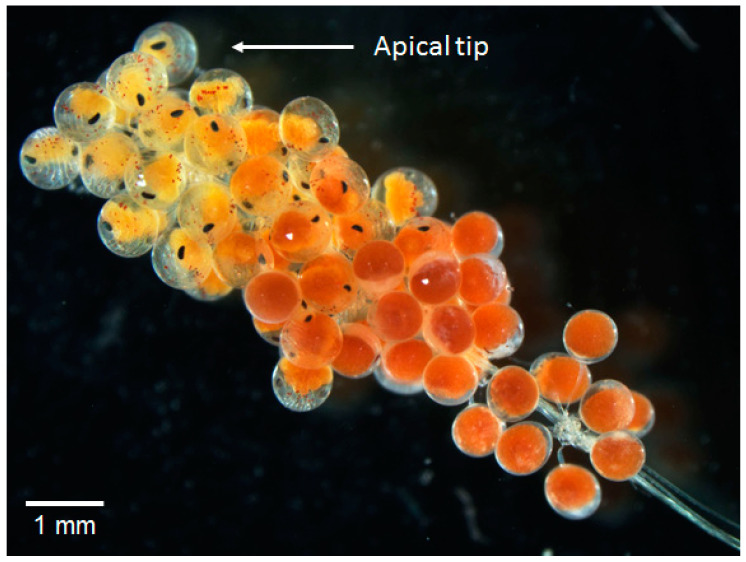
Dissection microscope picture of an ovigerous seta. Note the difference in developmental stage along the seta from R to L.

**Figure 3:**
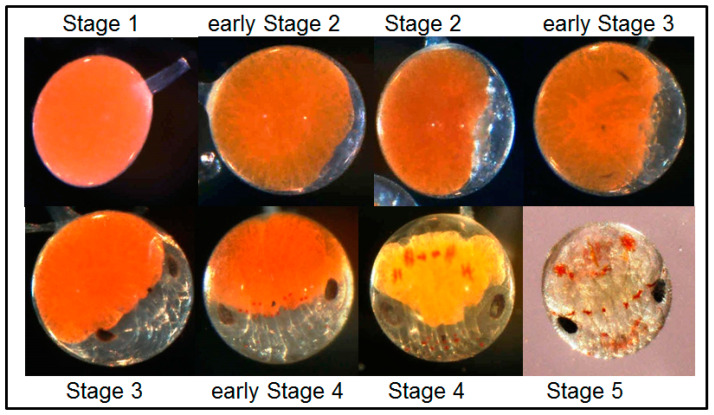
Staging index used for determination of developmental stages of fertilised eggs (own reference pictures staged according to Silberbauer [19].

For examination of developmental stages of embryos, two ovigerous setae were separated from the fourth pair of pleopods of female lobsters at each sampling time point. Except for the initial sampling (45–60 eggs), from each of these setae, 25 to 30 eggs were gently removed, starting from the apical tip, because egg development was not homogenous along a seta, but apical egg development is more rapid (Figure 2). This procedure was carried out at the start of the incubation (day 0) and repeated every 10 days until the embryos hatched (day 30 for Knol eggs, day 50 for Olifantsbos eggs). Embryonic development stage (Figure 3) was determined according to Silberbauer [19] using a light microscope (SMZI 1500, Nikon, Tokyo, Japan) equipped with a digital camera (DSFI 1, Nikon, Japan). From the same pictures, egg size (diameter) was analysed by NIS software Ver5.22.00 for Windows (basic research package BR, Nikon Imaging Systems, Japan). Since no females were caught with eggs at stage 1, embryonic development was observed using females whose eggs were at stage 2 of the scale (Figure 3). Berried setae were also analysed for fungus-infected or decaying eggs.

### 2.6. Hepatosomatic Index (HSI) and Gonadosomatic Index (GSI)

Females used for analysing embryonic development were incubated for four more weeks after embryos had hatched. After their total body mass was measured, they were anaesthetised and then euthanised in a mixture of seawater and ice and dissected to measure the mass of gonads and the hepatopancreas. Values for GSI and HSI were calculated as described in Munian et al. [22]. Macroscopic analyses of gonad development were conducted according to Heydorn [12].

### 2.7. Scanning Electron Microscopy (SEM) Analysis

Samples were kept in formalin to analyse the surface of the egg membrane (chorion) using SEM. One seta of each female was later fixed in 10% formalin with a phosphate buffer and stored at room temperature. Dehydration of egg samples was accomplished through a graded acetone series from 35% to 100% according to Gil-Turnes and Fenical [34] and critical point drying (FL-9496, Blazer Union) using CO_2_ as an intermediate fluid was then carried out. A defined area of the 4th right pereiopod (*merus*), as well as a defined area (a triangle cut from the posterior end of the carapace), was cut from three animals of each incubation group, fixed in 10% formalin with a phosphate buffer, and stored at room temperature. To dehydrate samples, formalin was washed out using double-deionised water. Samples were then dried in ambient air to avoid artefacts through dehydration in a drying oven. Embryo and exoskeleton samples were sputter-coated with gold (Agar Sputter Coater) at 1 × 180 s and observed on a Leo 1430 VP scanning electron microscope.

### 2.8. Statistical Analysis

All data were tested for normality using the Shapiro–Wilk test and for homogeneity of variance using the Brown–Forsythe test. Statistics were determined using Sigma Plot version 14.0. The significance level chosen throughout was *p* < 0.05. Results of acute exposure were analysed by two-way ANOVA, followed by post hoc Dunnett’s test to test for differences from initial value (t_0_) within treatments and between time pairs of treatments. GSI, HSI, acid–base parameters, and haemolymph data of the chronic exposure were analysed by one-way ANOVA followed by post hoc Tukey test. Egg growth rates during chronic exposure were analysed by two-way ANOVA followed by post hoc Holm–Šidák test.

## 3. Results

### 3.1. Acute Experiments—Acid Base Regulation

**Figure 4:**
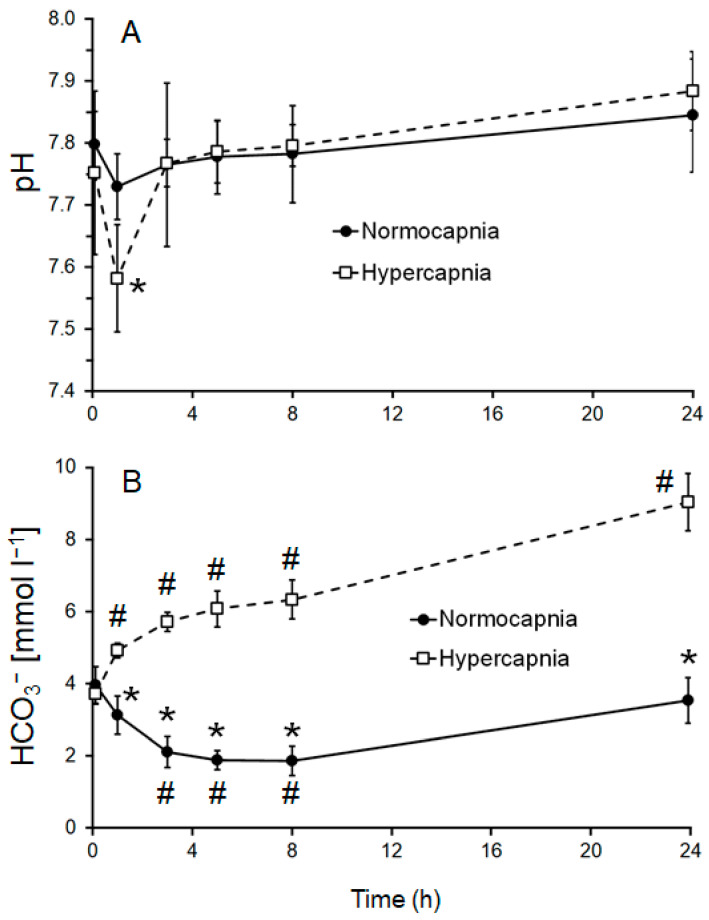
Time course of acid–base parameters in haemolymph of berried *J. lalandii* from the Olifantsbos area: (**A**) measured pH and (**B**) calculated [HCO_3_^−^ + CO_3_^2−^] during acute exposure to normocapnic seawater and hypercapnia for 24 h. Values are means ± S.D. # Significant difference from initial value (t_0_) within the treatment; * significant difference from the same respective sampling time of the normocapnic treatment (repeated-measure ANOVA; *p* < 0.05).

**Figure 5:**
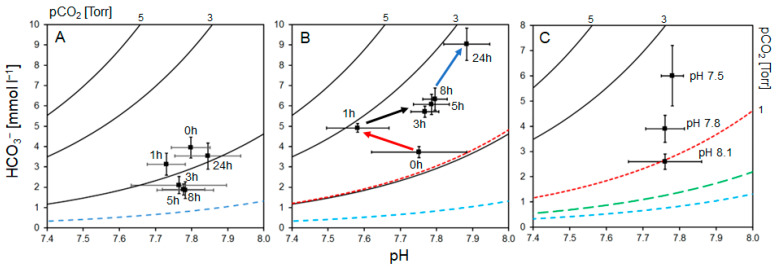
Henderson–Hasselbalch (pH–bicarbonate) diagrams for haemolymph of berried *J. lalandii* from the Knol area constructed from the time course of values during acute exposure presented in Table 2 and after experimental incubation for 60 days: (**A**) during 24 h normocapnia, (**B**) during 24 h hypercapnia (pH = 7.5), and (**C**) during incubation for 60 days (data from Table 3). pCO_2_ isopleths were derived from the Henderson–Hasselbalch equation [9]. Appropriate values for the first dissociation constant (pK′1) and solubility coefficient (α) were derived from Truchot [33]. Values are means ± S.D. Blue dashed line = normocapnic seawater isopleth, red dashed line = hypercapnic seawater isopleth (pH = 7.5), green dashed line = hypercapnic seawater isopleth (pH = 7.8). Arrows indicate in (**B**) course of bicarbonate buffering from start to 24 h hypercapnic incubation (red = acidosis, blue = alkalosis).

Throughout the acute exposure, haemolymph parameters showed different patterns of variation under normocapnic and hypercapnic conditions: In the normocapnic group, haemolymph pH decreased marginally by 0.06 units within the first hour. From then on, pH increased steadily during the next 3 to 8 h and reached a pH of 7.85 after 24 h (Figure 4A, Table 2). Extracellular total CO_2_ (cCO_2_) levels declined steadily to a low of 1.9 mmol L^−1^ after 3 to 5 h of exposure. Subsequently, total CO_2_ concentration increased to reach 3.6 mmol L^−1^ after 24 h (Table 2). The calculated bicarbonate + dissolved CO_2_ concentration, [HCO_3_^−^ + CO_3_^2−^], hereafter called “bicarbonate” for simplicity, declined steadily to a low of 1.9 mmol L^−1^ after 3 to 5 h of exposure. Subsequently, concentration increased to 3.5 mmol L^−1^ after 24 h (Figure 4B, Table 2). A similar course of changes was detected for pCO_2_ values (Table 2). The Henderson–Hasselbalch diagram of the normocapnic treatment showed very little change in bicarbonate levels, pCO_2_, and pH (Figure 5A).

In contrast, following an ambient pH decrease of 0.5 units, a substantial decrease in extracellular pH by 0.17 units was measured after 1 h of exposure, followed by a steep increase to pH 7.77 after 3 h. Subsequently, there was a gradual and continuous increase to reach a final pH of 7.88 after 24 h (Table 2, Figure 4A). This is an overcompensation of pH by approximately 0.12 pH units or reduction in [H^+^] by 5 nM (28%) compared with initial levels. Extracellular total CO_2_ (cCO_2_) levels increased steadily throughout 24 h of exposure (Table 2). Calculated bicarbonate levels also steadily increased throughout the entire exposure (Table 2, Figure 4B). pCO_2_ values followed a similar trend (Table 2). Data were used to construct a Henderson–Hasselbalch diagram (Figure 5B). During the first hour of exposure to hypercapnia, a respiratory acidosis is apparent, indicated by a shift to the left (red arrow). Simultaneously, bicarbonate starts to increase, buffering the extracellular acidosis and reversing the acidosis after 3 h of exposure, indicated by a shift back to the right (black arrow). Subsequently, the continued bicarbonate increase leads to an alkalosis when compared to the initial pH. This is indicated by a shift further to the right (blue arrow). Levels of molecular modulators of haemocyanin oxygen affinity, Ca_2_^+^, Mg_2_^+^, and L-lactate did not change during the course of the experiment and did not differ between treatments (Table 2). Protein concentration dropped steadily in both treatments until the end of experimentation (Table 2). Simultaneously, protein and haemocyanin concentration steadily declined in both treatments to reach nearly half the initial level after 24 h (Table 2), probably due to the serial sampling of the animals.

### 3.2. Chronic Incubation—Acid–Base Regulation

After 60 days of chronic hypercapnic exposure at pH = 7.8 and pH = 7.5, haemolymph chemistry was analysed and compared with normocapnic exposure of pH = 8.1. Extracellular (haemolymph) pH was very similar in all treatments at a level of pH = 7.76 to 7.78 (Table 3, Figure 5C). The level of extracellular total CO_2_ (cCO_2_) was lowest in the normocapnic treatment at 2.7 ± 0.3 mmol L^−1^, whereas 4.0 ± 0.5 mmol L^−1^ was measured at pH = 7.8 treatment and higher in the pH = 7.5 treatment at 6.1 ± 1.2 mmol L^−1^. Calculated bicarbonate concentration was lowest in normocapnia at 2.6 ± 0.3 mmol L^−1^, whereas 3.9 ± 0.5 mmol L^−1^ was measured in pH = 7.8 hypercapnia and higher in pH = 7.5 hypercapnia at 6.1 ± 1.1 mmol L^−1^. Levels of pCO_2_ changed similarly to bicarbonate levels (Table 3). The Henderson–Hasselbalch diagram revealed that at similar pH in lobsters from all treatments, elevation of pCO_2_ and bicarbonate caused vertical shifts in the haemolymph buffer lines in lobsters incubated at two levels of hypercapnia, with those from the pH = 7.5 treatment highest (Figure 5C). This indicated bicarbonate buffering that resulted in alkalosis in the hypercapnic groups. Both pCO_2_ and bicarbonate were significantly higher compared to the normocapnic group as well as within the two hypercapnic treatments, where the pH 7.5 treatment yielded a 55% higher bicarbonate level than the pH 7.8 treatment. At the same time, there were no changes in absolute protein concentration throughout the 60-day incubation for any treatment, but there were increases in the haemocyanin concentration from normocapnic levels of 11.9 mg ml L^−1^ to 14.6 and 16.5 mg L^−1^ at pH 7.5 and 7.8, respectively.

### 3.3. Chronic Exposure—Embryonic Development

**Figure 6:**
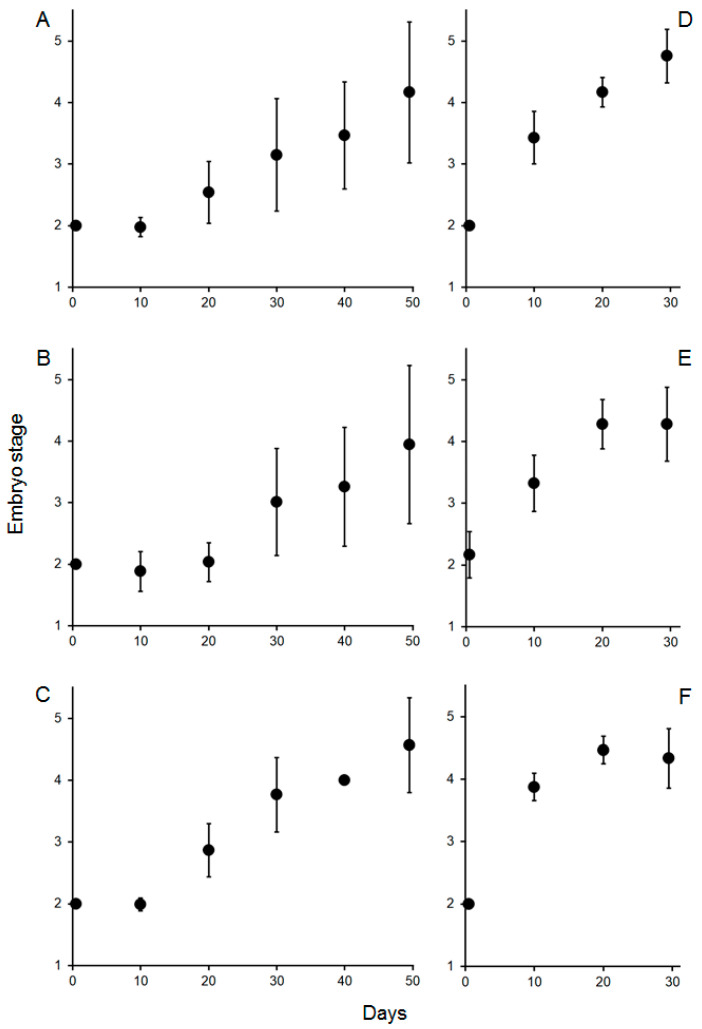
Mean (± S.D.) developmental stage of embryos [18] from berried lobsters from the Olifantsbos (n = 676; left column of figures) and Knol areas (n = 680; right column of figures) during 30 or 50 days of incubation. (**A**,**D**) Normocapnia, (**B**,**E**) pH 7.8, (**C**,**F**) pH 7.5. Note: x-axes of left and right panels are of the same scale to ensure comparability.

Embryonic development was monitored by staging and size measurement of eggs. Initial embryo stage [19] was from 2.0 to 2.2 for eggs from females from the Knol area and 2.0 for eggs from females from Olifantsbos (Figure 6). At the end of the exposure, developmental stages were between 4.3 and 4.8 in the treatments of eggs from the Knol area (30 days of incubation), whereas they were between 3.9 and 4.2 in the treatments from Olifantsbos females after 50 days (Figure 6). Due to the non-linearity of the results and uncertainties described (see comments in Discussion), a statistical analysis was omitted.

**Figure 7:**
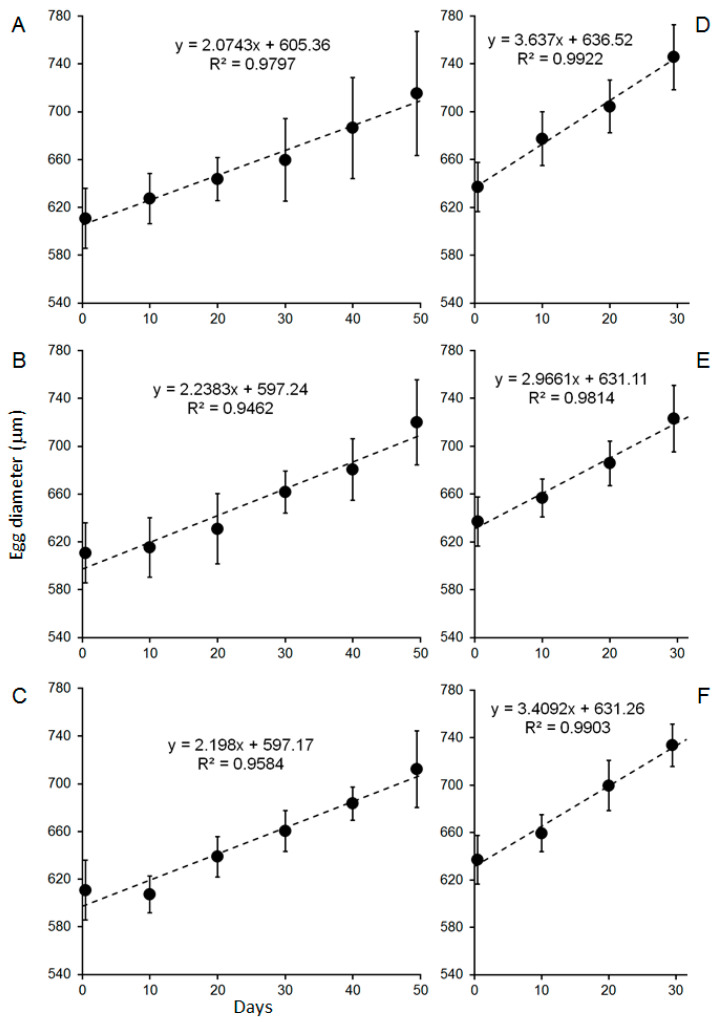
Egg growth, expressed as mean (± S.D.) diameter, in berried lobsters from the Olifantsbos (n = 676; left column of figures) and Knol areas (n = 680; right column of figures) during 50 and 30 days of incubation, respectively. (**A**,**D**) Normocapnia, (**B**,**E**) pH 7.8, (**C**,**F**) pH 7.5. Note: x-axes of left and right panels are of the same scale to ensure comparability.

**Figure 8:**
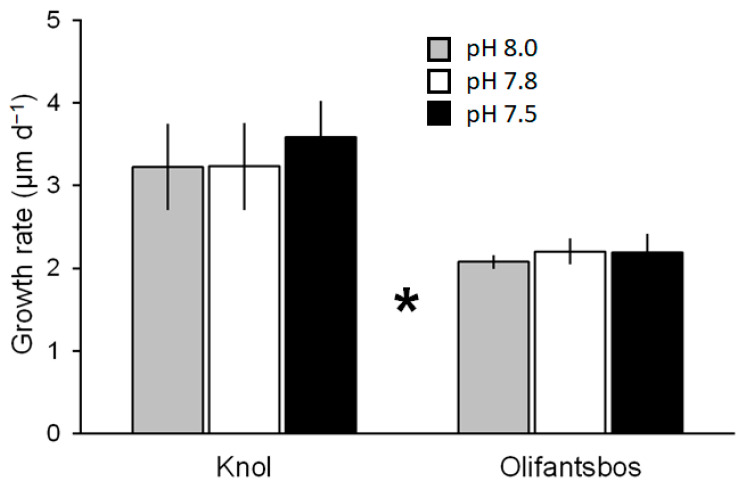
Daily growth rate (µm d^−1^) of embryos/eggs from berried rock lobsters from the Knol and Olifantsbos areas under three pH treatments (see Table 1) calculated from correlation slopes of egg growth from individual females (see Figure 7). * Significant difference between locations (*p* < 0.001, two-way ANOVA and post hoc Holm–Šidák test).

Initial egg diameters were approximately 637 ± 21 µm (n = 680) for eggs from Knol females, whereas they were 612 ± 25 µm (n = 676) for those from Olifantsbos females (Figure 7). After incubation of 30 days, diameters ranged from 724 to 746 µm in treatments of Knol embryos and from 712 to 720 µm after 51 days of incubation from treatments of Olifantsbos embryos (Figure 7). Growth in egg size was linear in all treatments of eggs from both sampling areas (Figure 7). Growth rates ranged from 3.2 to 3.6 µm d^−1^ in treatments of embryos from the Knol area and from 2.1 to 2.2 µm d^−1^ in treatments of those from the Olifantsbos area (Figure 8). There was no significant difference between treatments within the respective areas. However, growth rates of Knol eggs from all treatments were 47–64% higher than those from all treatments of the Olifantsbos area (Figure 8). No fungal infected or decayed eggs/embryos were observed during sampling.

### 3.4. Chronic Exposure—Gonadosomatic and Hepatosomatic Index

Females from the Olifantsbos area had lower GSIs than females from the Knol area. The GSI of the Olifantsbos normocapnic group was calculated at 0.7 ± 0.2% (mean ± S.D., n = 12). A significantly lower GSI was estimated for the pH 7.8 treatment—0.3% ± 0.1%. The pH 7.5 treatment was not significantly different from hypercapnia, but was slightly lower, at 0.4 ± 0.2%. Hypercapnic lobsters from the Knol area had a GSI of 0.85% ± 0.6%. The pH 7.8 and 7.5 treatments, were similar with 0.6 ± 0.1% and 0.5 ± 0.1%, respectively, both lower than the normocapnic treatment. Normocapnic treatments had higher GSIs compared with the respective hypercapnic treatments. The HSI values of normocapnic (pH = 8.0) females from the Olifantsbos area were 1.9 ± 0.3% (mean ± S.D., n = 12), whereas those in the hypercapnic treatments were 2.1 ± 0.5% and 1.9 ± 0.3%, respectively. Normocapnic females from the Knol area had a lower HSI at 2.2 ± 0.1% than both hypercapnic treatments at 2.4 ± 0.4%. However, this difference was not significant.

### 3.5. Chronic Exposure—Structural SEM Analysis of the Cuticle and Eggs

**Figure 9:**
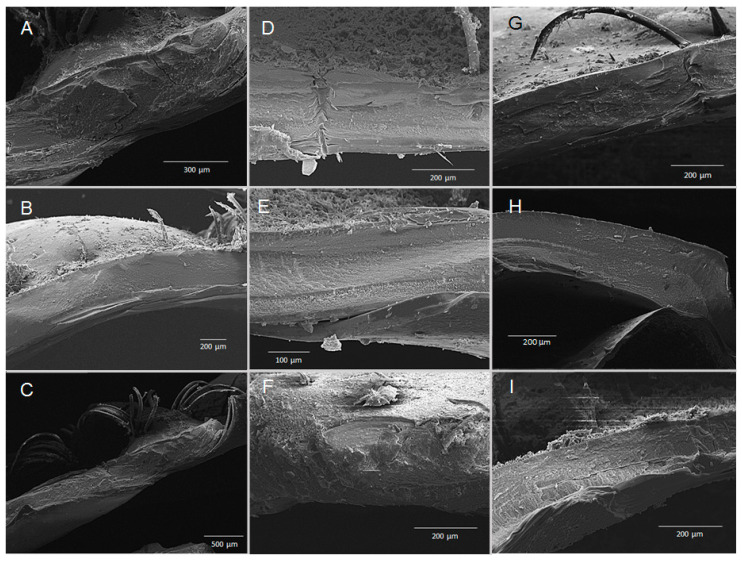
SEM micrographs of transversally fractured/cross-fractured cuticle of a defined *merus* sample each from individual berried *J. lalandii* from the Olifantsbos area after experimental exposure of 50 days. (**A**–**C**) Normocapnia, (**D**–**F**) pH 7.8, (**G**–**I**) pH 7.5. Scale bars are given in individual panels.

**Figure 10:**
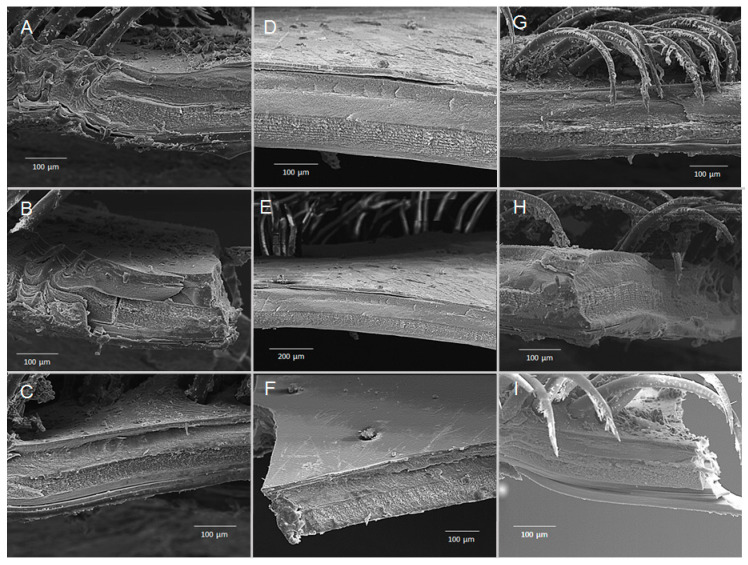
SEM micrographs of a transversally fractured/cross-fractured cuticle from a defined carapace area from individual berried *J. lalandii* from the Olifantsbos area after experimental exposure of 50 days. (**A**–**C**) Normocapnia, (**D**–**F**) pH 7.8, (**G**–**I**) pH 7.5. Scale bars are given in individual panels.

**Figure 11:**
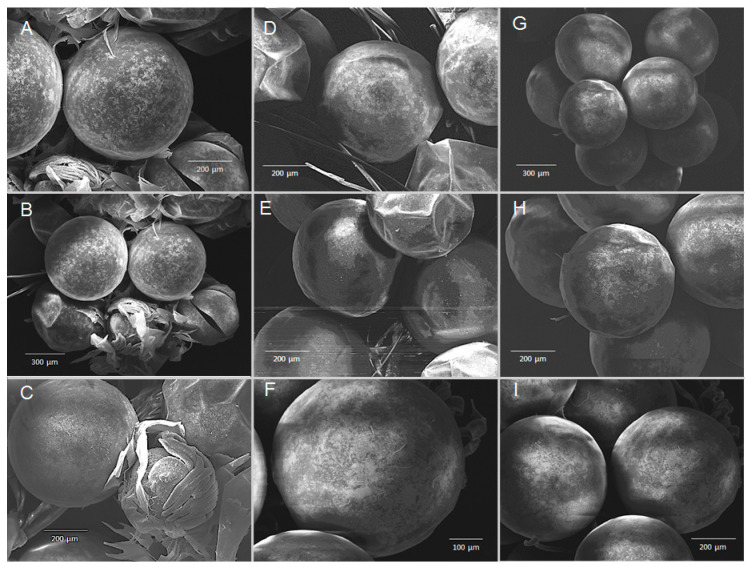
SEM micrographs of egg membranes (chorion) from individual berried *J. lalandii* from the Olifantsbos area after experimental exposure of 50 days. (**A**–**C**) Normocapnia, (**D**–**F**) pH 7.8, (**G**–**I**) pH 7.5. Scale bars are given in individual panels.

Scanning electron microscopic (SEM) analysis of transversally fractured/cross-fractured cuticle from *merus* samples revealed no obvious abnormalities or structural damages that could have derived from chronic hypercapnic treatments, such as decalcification of the cuticle layers (Figure 9). Similarly, carapace samples had no obvious damage or abnormalities (Figure 10). Hair-like structures in some panels of Figure 8 and Figure 9 depict setae that are rooted in the cuticle and partially cover the exoskeleton. SEM analysis of the embryonic cuticle (chorion) of eggs from all treatments at the end of experimentation did not reveal structural differences or damage to the surface, such as cracks or dissolution of the egg membrane itself (Figure 11). Folding and dents or depressions are artefacts of the sample treatment process for SEM determinations.

## 4. Discussion

We previously reported that mature males and juveniles of the WCRL are resilient to acute and chronic hypercapnia [9,10,11]. Our present results extend this observation for the first time to ovigerous females and their embryos according to comparable criteria.

This is apparent in the regulation of acid–base balance during acute hypercapnia: as in mature males of similar size [10], berried females adjust their acid–base balance rapidly by means of bicarbonate, which is elevated by a net 5.2 mmol L^−1^ (+133%) after 24 h. This increase in haemolymph bicarbonate levels in response to a pH = 7.5 is even more pronounced than that of adult male WCRLs after acute hypercapnia at a lower pH of 7.3 [10]. Bicarbonate buffering is a common response in crustaceans (mainly crabs) to respiratory or extracellular acidosis [27,35,36,37,38,39]. It is also required to compensate for disturbance of acid–base balance to achieve resting pH levels [38] or close to original values [35,36,37], i.e., within the physiological range. When exposed to an ambient pH of 7.5, ovigerous WCRLs fully compensated for the decreased environmental pH within 3 h post-exposure (Figure 4A, Table 2). This compensation is rapid and complete when compared with other crustacean species, but similar to male WCRLs [10]. A pronounced Bohr effect was measured in juvenile [9] and mature male WCRLs [10]. Therefore, oxygen affinity needs to be safeguarded to ensure oxygen loading of haemocyanin at the gills and offloading at the tissues. This is accomplished by the above-mentioned bicarbonate buffering of their haemolymph to provide optimum pH conditions. Bicarbonate buffering also guarantees an outward gradient for metabolic CO_2_ removal by maintaining the extracellular pCO_2_ above environmental pCO_2_. Here, the gradient after 24 h hypercapnic incubation is 3.5 Torr, whereas it is only 0.9 Torr under normocapnia.

The initial prebranchial extracellular pH for ovigerous *J. lalandii* throughout experiments was approximately 7.8 (T_A_ range: 14–17 °C, Table 2 and Table 3), i.e., approximately 0.2 pH units below ambient pH. This is similar to adult male WCRLs [10]. During acute exposure to a pH of 7.5, berried lobsters reached a minimum extracellular pH after 1 h, which was 44% lower (acidosis) than the initial value (Figure 4A, Table 2). During previous work on mature male *J. lalandii*, however, a strong respiratory acidosis set in during the first 25 min of hypercapnia at pH = 7.4 before bicarbonate buffering took effect [10]. This is indicative of a transient acidosis. At the 1 h sampling point, this acidosis was most likely already partially compensated by buffering in berried females. Additionally, it may have been masked by an alkalosis caused by hyperventilation [40]. This effect was considered small, however, as judged by the stable lactate concentration (Table 2).

The Henderson–Hasselbalch diagrams provide insight into the interaction of extracellular pH, calculated haemolymph bicarbonate, and pCO_2_: In the WCRL group under normocapnic conditions, there is very little change compared with hypercapnic exposure (Figure 5A,B). The small changes observed, however, were caused by the changes in extracellular pCO_2_, pH, and bicarbonate (Table 2, Figure 5A), possibly as a result of handling stress. After exposure to hypercapnic conditions, there is a strong transitional acidosis within 1 h after which compensation by bicarbonate becomes effective (Figure 5B). After 3 h of exposure, compensation by increased bicarbonate levels (along a constant pCO_2_ isopleth) becomes apparent and the pH starts to increase (shift to the right). Subsequently, further increasing bicarbonate levels cause a move into an alkalosis and overcompensation (Figure 5B). This cause of events resembles that found in male adult WCRLs during hypercapnia [10].

The above acid–base regulation is still present after 60 days of chronic hypercapnia: berried WCRLs are still capable of bicarbonate buffering of their haemolymph to provide optimum pH conditions for oxygen binding in the presence of a strong Bohr effect (Figure 5C). This ensures functioning of respiration during prolonged hypercapnia and is similar to the response previously observed in juvenile WCRLs after chronic hypercapnia [9] and in other crustaceans [27,38,39]. After 24 h of acute hypercapnia, the extracellular pH was 0.2 units higher than ambient pH (Table 1 and Table 2). This differential was maintained during chronic hypercapnia (Table 1 and Table 3). However, the high outward pCO_2_ gradient found after acute hypercapnia (see above) was reduced to 1.0–1.2 Torr, not much above the respective normocapnic gradient of 0.7 Torr (Table 1 and Table 3). This suggests that the high gradient in the acute experiment was because of overcompensation for the sudden increase in environmental pCO_2_. This overcompensation, causing alkalosis in the acute experiment, is therefore not maintained (Figure 5C). Acid–base regulation is associated with high energetic costs [25,41,42]. It is therefore possible that other metabolic functions of the animal are reduced to make this additional energy available [25]. One of those functions is maternal care. This means aerating the eggs by lifting the abdomen, fanning with the pleopods, and grooming the eggs with a special appendage on the fifth pair of pereiopods [19,43,44]. The latter specifically prevents fouling of the eggs in ovigerous *Jasus* females by removing fungus infected or decaying eggs and therefore improves general survival [45]. Fanning and lifting of the abdomen provide oxygenation of eggs, especially those that are located away from the apical tip of setae. Oxygen supply of eggs is critical for their development and survivorship, as shown for crabs [46]. Also, in crab species (*Ovalipes trimaculatus, Paralithodes camtschaticus*), brood care was shown to increase energetic cost to the brooding female by more than 100% of the measured oxygen consumption [47,48]. This is in addition to the cost of acid–base regulation during hypercapnia (see above). Successful growth and development of embryos as well as hatching of larvae from all treatments suggests that essential maternal care was not impaired by chronic hypercapnia (Figure 6, Figure 7 and Figure 8). Moreover, no fungus-infected, fouled, or decaying eggs were observed during egg sampling, probably because the females may have removed them. Therefore, successful growth, development, and hatching of embryos and a constant extracellular pH suggest that the females can balance the sum of costs for maternal care and acid–base regulation.

It is generally assumed that early life stages of marine invertebrates are the most vulnerable life stage to ocean acidification [23]. However, there are extremely limited data to support this statement for larvae and embryos of decapods, mostly due to a lack of research [7,25,27]. A recent meta-analysis [27] and the few publications available demonstrate low or no impact on early life stages from other decapod crustaceans. Examples are crab eggs (*Leptuca thayeri*) that were resilient to very low pH during very short development (and incubation) time of 10 days [26]. Embryos of the snow crab (*Chionoecetes opilio*) were unaffected by hypercapnia of pH 7.8 and 7.5 during development/incubation periods of up to two years at very low temperatures (~2 °C) [8]. Larvae of another crab species, the red king crab (*Paralithodes camtschaticus*), are resilient in terms of survival and development to simulated ocean acidification of almost two years in length [48]. Moreover, *Nephrops norvegicus* embryos also were found to be insensitive to low pH and elevated temperatures [49]. Such insensitivity of embryos to low pH might be explained by adaptation to a pH-reduced external habitat and/or internal hypercapnia during incubation [49]. In contrast, there are several studies that do reveal sensitivities of decapod embryos and larvae to hypercapnia. For example, low pH levels decreased larval development rates in crustacean species, such as the barnacle *Semibalanus balanoides*, the crabs *Hyas araneus* and *Chionoecetes bairdi*, as well as the lobster *Homarus americanus* [50,51,52,53,54]. Larvae hatched from embryos of the red king crab *Paralithodes camtschaticus* that were incubated in hypercapnia (pH = 7.7) had a reduced survival rate when starved [55]. Research results from the present study place the WCRLs into the hypercapnia-resistant group of crustaceans: they revealed a relative resilience of egg development and growth against different levels of hypercapnia.

Previously, there were no data available on the impact of hypercapnia on palinurid embryos, whereas temperature effects were well studied, showing that embryo development accelerates with increasing temperature (for example, in *J. edwardsii*; [56,57]). We could close this knowledge gap somewhat by showing that development and growth of eggs/embryos are insensitive to hypercapnia: data from egg size measurements show that growth in diameter is linear for eggs from both collection sites and for all treatments (Figure 6). Because of this linearity and less subjectivity than egg development staging (see below), growth rates were used for further statistical analysis. The latter revealed no differences between the treatments for either location at which berried females were caught (Figure 8), indicated by similar slopes within charts for a respective catch location (Figure 7). Moreover, SEM analysis of eggs revealed no surface damage or differences between treatments (Figure 11). In contrast to measured egg diameters, results from developmental stages must be evaluated with caution. This is due to the more subjective, and potentially biased, nature of staging and non-linearity of results (Figure 6). In addition, the seasonal biological cycle of adult WCRLs (moulting, mating, spawning) commences first at northern latitudes and gradually moves south. As a result, egg development in the more northern Knol area is slightly ahead of Olifantsbos, indicated by the more advanced stage 2 eggs at Knol. The consequence was a shorter incubation period of 30 vs. 50 days at the aquarium, since Knol embryos were closer to hatching. The faster development and growth (see below) rates of Knol eggs also contributed to the shorter exposure period. Egg stage data are therefore difficult to statistically analyse and deemed not useful to conclude on the potential impact of hypercapnia on embryonic development.

Our study revealed an additional, interesting aspect of the developing eggs: independently of treatment, egg growth rates differed significantly between the two sampling sites. Rates at Knol were about 1.0–1.4 µm d^−1^ higher than those from Olifantsbos, independently of treatment (Figure 8). This is a 47–64% faster growth rate. Temperature was shown to affect the duration of embryonic development in the closely related *J. edwardsii* [56,57]. However, incubation temperature (and all other conditions) was identical for eggs from both sampling sites in the present study and can be ruled out as factor for the growth differential. Even after the Knol embryos had already hatched, growth of the Olifantsbos embryos remained linear under those conditions. The reason for the differential growth rate could therefore rather reflect the exposure of the mother in its environment before capture. The growth advantage of Knol embryos therefore carries over the favourable conditions experienced by Knol females: The larger diameter of eggs from Knol lobsters at the time of catch are most likely due to the above-mentioned advanced progress into stage 2 compared with those from Olifantsbos females. However, there may also be larger eggs for females from that area due to better growth conditions for adult lobsters. The difference in male somatic growth rates and female fecundity is well known for the two areas [16]. These differences have been shown to persist in recent years in terms of male somatic growth rates [58] and in female GSI [22]. Higher fecundity is concluded from the larger number of eggs. However, eggs may also be larger in high-fecundity areas. Consequently, larger eggs in the Knol area could be the result of favourable ambient conditions for parent lobsters. Since physicochemical conditions in the Knol and Olifantsbos areas are very similar, it is assumed that food availability and composition cause such favourable conditions [22]. Egg size differences at spawning, however, are so far not reported and will need future analysis.

In decapods, the response of exoskeleton calcification to hypercapnia is species-specific and depends on duration and severity of hypercapnia [27,59,60]. In addition, it can be specific to the body region [61]. Therefore, samples from two different areas of the WCRL exoskeleton were analysed by SEM in the present study. Neither surfaces nor structures of merus and carapace pieces revealed any visible differences between treatment groups (Figure 9 and Figure 10). This was different from a similar hypercapnic experiment where the surface of different exoskeleton parts of the *Cancer pagurus* crab became porous and small cavities developed during hypercapnia. In addition, cracks developed between exocuticle and endocuticle [62]. It must be noted, however, that although the incubation period was similarly short (60 and 56 days, respectively), the crabs were exposed to a lower hypercapnia at pH 7.35 than the WCRLs (pH 7.5 and 7.8, respectively). In both cases, the experimental period did not include a moult. A developing new exoskeleton would probably be differently affected than an existing old and calcified one [25]. This relative resilience of the exoskeleton is not surprising, since crustaceans are well capable of maintaining or increasing the calcification rate during exposure to acidified water [25,27,38]. Moreover, in the decapod exoskeleton, calcium carbonate is embedded in its amorphous form into a matrix of chitin–protein fibres [61,63]. It is therefore not as exposed to ambient conditions as, for example, mollusc shells and sea urchin tests are. However, maintenance of this structure may have consequences for energy expenditure and/or diversion from other key biological aspects if extended for longer periods [25,64,65,66,67]. The present study was not designed to investigate this aspect.

Reproductive organ size was analysed at the end of the incubation for a few females in the present study. Both HSI and GSI values were higher in Knol females. This is similar to a previous study in which many more female WCRLs were analysed [22]. Moreover, at ovary stage 5, ovaries from Knol females were relatively larger than those from Olifantsbos area [21]. The growth differences of eggs reported here (Figure 8) are in agreement with this, and may point to better maternal transfer from females to eggs in the Knol area. Therefore, larger ovaries of lobsters from Hout Bay may have resulted in larger eggs with larger reserves, leading to a development advantage over those from Olifantsbos females. This hypothesis is difficult to prove in the field and needs further analysis.

## 5. Conclusions

Our study has shown that ovigerous female WCRLs regulate acid–base balance similarly to males and juveniles in response to acute and chronic hypercapnia. Moreover, embryonic development was resilient to prolonged hypercapnia. Differences in development occurred, however, between the two sampling areas of the respective ovigerous females. This is in line with historic differences in male somatic growth and female fecundity between these two distinct geographical areas, which are assumed to be caused by differences in food availability and composition.

## Figures and Tables

**Table 1:** Physicochemical seawater conditions recorded during exposure of ovigerous *J. lalandii* to normocapnic and hypercapnic conditions.

Treatment	T_A_ °C	pH	A_T_ µmol kg^−1^	O_2_ %	Salinity ‰	Ca^2+^ mmol L^−1^	Mg^2+ ^ mmol L^−1^	pCO_2_		HCO_3_^−^ mmol L^−1^	CO_3_^2−^ mmol L^−1^
								Torr	(µatm)		
*Acclimation*	14.2 ± 0.0	7.93 ± 0.02	2001 ± 21	97.1 ± 0.8	34.9 ± 00	10.1 ± 0.3	52.0 ± 1.9	0.3 ± 0.0	(460 ± 26)	1.7 ± 0.0	0.1 ± 0.0
*Acute exposure*											
Normocapnia	14.0 ± 0.2	7.95 ± 0.04	1997 ± 24	96.0 ± 1.4	34.9 ± 0.1	10.2 ± 0.6	50.5 ± 2.7	0.3 ± 0.0	(443 ± 37)	1.7 ± 0.0	0.1 ± 0.0
Hypercapnia 7.5	14.1 ± 0.3	7.47 ± 0.07	2003 ± 15	95.4 ± 0.8	34.9 ± 0.2	10.0 ± 0.2	51.2 ± 3.8	1.1 ±0.2	(1463 ± 236)	1.9 ± 0.0	0.0 ± 0.0
*Chronic exposure*											
Normocapnia	17.1 ± 1.4	7.99 ± 0.10	2034 ± 40	93.6 ± 1.5	35.0 ± 0.0	10.3 ± 0.6	52.8 ± 1.9	0.3 ± 0.1	(410 ± 130)	1.7 ± 0.1	0.1 ± 0.0
Hypercapnia 7.8	17.2 ± 1.2	7.80 ± 0.01	2029 ± 42	92.4 ± 1.4	35.0 ± 0.0	10.2 ± 0.5	52.4 ± 1.5	0.5 ± 0.0	(665 ± 25)	1.8 ± 0.0	0.1 ± 0.0
Hypercapnia 7.5	16.9 ± 1.1	7.52 ± 0.04	2032± 42	94.6 ± 1.8	35.0 ± 0.0	10.2 ± 0.3	52.6 ± 1.3	1.0 ± 0.1	(1338 ± 138)	1.9 ± 0.0	0.1 ± 0.0

**Table 2:** In vivo haemolymph parameters of ovigerous *J. lalandii* from the Olifantsbos area after acute exposure to normocapnic and hypercapnic seawater conditions.

Exposure Time (h)	pH	cCO_2_ mmol L^−1^	pCO_2_		[HCO_3_^−^ + CO_3_^2−^] mmol L^−1^	Ca^2+^ mmol L^−1^	Mg^2+^ mmol L^−1^	Protein mg mL^−1^	Haemocyanin mg mL^−1^	L-lactate mmol L^−1^
			Torr	µatm						
Normocapia										
0	7.79 ± 0.05	4.0 ± 0.5	1.5 ± 0.4	2632 ± 395	4.0 ± 0.5	15.6 ± 1.7	7.9 ± 0.2	28.9 ± 3.9	20.0 ± 3.9	0.2 ± 0.1
1	7.73 ± 0.05	3.2 ± 0.5 #	1.3 ± 0.1	2105 ± 394	3.1 ± 0.5	15.7 ± 2.5	8.1 ± 1.5	29.0 ± 5.8	19.7 ± 5.2	0.3 ± 0.1
3	7.77 ± 0.13	2.1 ± 0.4 #	0.9 ± 0.2	1447 ± 263	2.1 ± 0.4 #	15.4 ± 2.3	8.9 ± 0.8	27.7 ± 5.4	18.0 ± 5.4	0.2 ± 0.1
5	7.78 ± 0.06	1.9 ± 0.3 #	0.7 ± 0.1 #	1316 ± 132	1.9 ± 0.3 #	14.4 ± 1.4	8.0 ± 1.4	23.3 ± 2.1	13.2 ± 1.1	0.2 ± 0.1
8	7.78 ± 0.08	1.9 ± 0.4 #	0.7 ± 0.0	1184 ± 264	1.9 ± 0.4 #	13.0 ± 2.6	9.7 ± 0.6	20.9 ± 4.4	9.8 ± 4.0	0.1 ± 0.1
24	7.85 ± 0.09	3.6 ± 0.7 #	1.2 ± 0.5	2368 ± 262	3.5 ± 0.6	16.1 ± 1.1	7.7 ± 0.8	22.3 ± 2.8	10.7 ± 2.6	0.2 ± 0.1
Hypercapnia										
0	7.76 ± 0.13	3.8 ± 0.3	1.6 ± 0.2	2634 ± 260	3.7 ± 0.3	12.9 ± 0.8	9.1 ± 1.2	29.6 ± 7.0	18.5 ± 7.7	0.4 ± 0.2
1	7.58 ± 0.09 *	5.1 ± 0.2 *	2.5 ± 0.1	3289 ± 132	4.9 ± 0.2 #*	11.6 ± 0.9	9.6 ± 1.0	30.2 ± 8.1	19.2 ± 9.0	0.4 ± 0.1
3	7.77 ± 0.04	5.8 ± 0.3 #*	2.9 ± 0.1 #*	3816 ± 136	5.7 ± 0.3 #*	12.7 ± 3.0	10.3 ± 0.6	28.7 ± 4.9	16.7 ± 4.2	0.3 ± 0.1
5	7.79 ± 0.05	6.2 ± 0.5 #*	3.0 ± 0.3 #*	3947 ± 398	6.1 ± 0.5 #*	10.5 ± 2.2	10.6 ± 0.9	25.3 ± 4.3	15.1 ± 6.2	0.3 ± 0.2
8	7.80 ± 0.03	6.4 ± 0.5 #*	3.1 ± 0.3 #*	4079 ± 391	6.3 ± 0.5 #*	12.0 ± 2.4	11.8 ± 0.7	23.6 ± 2.6	12.3 ± 2.5	0.3 ± 0.2
24	7.88 ± 0.06	9.1 ± 0.8 #*	4.6 ± 0.3 #*	6053 ± 393	9.0 ± 0.8 #*	11.7 ± 3.3	9.2 ± 1.2	23.1 ± 3.1	12.0 ± 2.7	0.2 ± 0.2

Values are means ± S.D. (n = 4–5). # Significant difference from the initial value (t_0_) within treatment; * significant difference from the same respective sampling time of the normocapnic treatment (repeated-measure ANOVA; *p* < 0.05).

**Table 3:** In vivo haemolymph parameters of ovigerous *J. lalandii* from the Knol area after chronic exposure to normocapnic and hypercapnic seawater conditions for 60 days.

Exposure Time	pH	cCO_2_ mmol L^−1^	pCO_2_		[HCO_3_^−^ + CO_3_^2−^] mmol L^−1^	Ca^2+^ mmol L^−1^	Mg^2+^ mmol·L^−1^	Protein mg mL^−1^	Haemocyanin mg mL^−1^	L-lactate mmol L^−1^
			Torr	µatm						
Normocapnia	7.76 ± 0.10	2.7 ± 0.3	1.0 ± 0.3	1316 ± 395	2.6 ± 0.3	12.9 ± 2.1	9.5 ± 1.4	23.5 ± 3.6	11.9 ± 4.1	0.2 ± 0.1
Hypercapnia pH 7.8	7.76 ± 0.05	4.0 ± 0.5 *	1.5 ± 0.2 *	1974 ± 263	3.9 ± 0.5 *	12.1 ± 1.7	10.2 ± 0.9	23.6 ± 3.9	14.6 ± 4.2	0.2 ± 0.1
Hypercapnia pH 7.5	7.78 ± 0.02	6.1 ± 1.2 *	2.2 ± 0.5 *	2632 ± 658	6.1 ± 1.1 *	14.8 ± 3.2	9.9 ± 1.2	24.2 ± 3.8	16.5 ± 2.9 *	0.1 ± 0.1 *

Values are means ± S.D. (n = 10). * Significant difference from the normocapnia treatment (one-way ANOVA; *p* < 0.05).

## Data Availability

Relevant information is included in the article. Raw data supporting the conclusions are available from the author, L.A., upon request.
